# Association of circulating angiogenesis inhibitors and asymmetric dimethyl arginine with coronary plaque burden

**DOI:** 10.1186/s13069-015-0029-6

**Published:** 2015-07-21

**Authors:** David M. Charytan, Angeles Cinelli, Elisabeth M. Zeisberg

**Affiliations:** Renal Division, Department of Medicine, Brigham and Women’s Hospital, Boston, MA USA; Departments of Cardiology and Pneumology, University Medical Center, Georg-August University, Robert-Koch-Str. 40, Göttingen, 37075 Germany; DZHK (German Centre for Cardiovascular Research), Partner Site Göttingen, Göttingen, Germany

## Abstract

**Background:**

Chronic kidney disease (CKD) is an independent risk factor for the development and severity of coronary artery disease (CHD) and endothelial dysfunction. There is an increase in the circulating angiogenesis inhibitors endostatin (END), thrombospondin-2 (TSP), angiopoietin-2 (ANG) and the nitric oxide (NO) inhibitor asymmetric dimethyl arginine (ADMA) in CKD patients. The aim of this study was to evaluate associations of the serum level of these factors and of the related angiogenesis inhibitor, endoglin (ENG), with burden of coronary atherosclerosis.

**Methods:**

One hundred twenty-two patients undergoing coronary angiography were recruited from the cardiac catheterization lab at a single center. The total burden of coronary plaque (mm^2^) and the presence of coronary collaterals were quantified using quantitative coronary angiography (QCA). Serum levels of angiogenesis inhibitors were measured by ELISA (ENG, END, and ANG), Luminex assay (TSP), or HLPC (ADMA), respectively. Associations with plaque burden and coronary collateral supply were analyzed in multi-variable linear and logistic regression models.

**Results:**

There was no significant association found between levels of circulating ADMA, ENG, END, ANG, or TSP and coronary plaque burden or collateral formation.

**Conclusions:**

Our findings suggest that associations of circulating END, ENG, TSP, and ANG with cardiovascular mortality are unlikely to be mediated via direct effects on coronary plaque formation or by inhibition of collateral formation. Whether associations of these factors with mortality are mediated via local concentrations, myocardial tissue, or intra-plaque expression of these factors or by an effect on plaque vulnerability merits additional investigation.

## Background

Despite dramatic therapeutic advances in the recent years, ischemic heart disease remains the leading cause of death worldwide [1]. Although traditional, “Framingham” risk factors are well-established contributors to the pathogenesis of coronary disease, and factors beyond hyperlipidemia, diabetes, and hypertension appear to play important roles in the development and progression of atherosclerosis [2]. Among the myriad non-traditional risk factors implicated, endothelial dysfunction and plaque angiogenesis have received increasing attention as contributors to the progression of coronary artery disease [3, 4].

Under normal conditions, the vascular endothelium plays a key role in maintaining homeostasis, and it functions to promote vasodilation, inhibit luminal and vascular wall coagulation, and prevent the proliferation of smooth muscle and foam cells. However, these functions may be perturbed, particularly when the bioavailability of nitric oxide (NO) is low, leading to a state favoring vasoconstriction, thrombosis, vascular smooth muscle cell proliferation, and the generation of atherosclerotic plaque [5]. In the setting of such endothelial dysfunction, imbalances of pro and anti-angiogenic factors contribute to generation of, hemorrhage-prone, immature capillaries within the vessel wall or within nascent plaques. Thus, the regulation of angiogenesis appears to be a key factor in the propagation of coronary artery disease and rupture of atherosclerotic plaque [3].

In addition to playing a direct role in endothelial homeostasis [5, 6], NO is also an important mediator of angiogenesis. It both induces secondary changes in the activity and concentration of several angiogenesis inhibitors and is, in turn, modulated by their presence [7–13]. Thus, both NO bioavailability and the concentration of the related inhibitors of angiogenesis are likely to be closely linked to the presence of atherosclerosis. Moreover, we have recently shown that asymmetric dimethyl arginine (ADMA), a key, competitive inhibitor of endothelial nitric oxide synthase, and related circulating angiogenesis inhibitors endostatin (END), thrombospondin-2 (TSP), and angiopoietin-2 (ANG) are increased in patients with chronic kidney disease, a patient population at increased risk for coronary artery disease. We therefore undertook this study in order to analyze the associations of ADMA [14] and related circulating angiogenesis inhibitors with the burden of coronary atherosclerosis as measured using quantitative coronary angiography (QCA).

## Results

### Baseline characteristics and angiographic characteristics

There were 122 subjects who met the inclusion criteria and were enrolled (Table 1). The mean age was 61.4 ± 11.7 years. The majority of subjects (77.9 %) had hypertension, 37.5 % had diabetes, and 34.4 % had stage 3 or higher chronic kidney disease including eight subjects on dialysis. Prior myocardial infarction (MI) was commonly present in 29.5 % of subjects, while 35.2 % underwent angiography during an admission for acute coronary syndrome. The majority of subjects had one vessel disease, but 34 (27.9 %) had multi-vessel atherosclerosis.

**Table 1:** Baseline characteristics of the study population

Characteristic (*n* = 122)	Value (*N* (%))
Demographics	
Age (years), mean ± SD	61.35 ± 11.72
Male	82 (67.2)
Black	11 (9.0)
Blood pressure (mmHg), mean ± SD	
Systolic blood pressure	127.97 ± 23.01
	(*n* = 118)
Diastolic blood pressure	70.19 ± 13.29
	(*n* = 118)
Medical history	
Acute coronary syndrome on presentation	43 (35.2)
Prior myocardial infarction	36 (29.5)
Hypertension	95 (77.9)
Diabetes	46 (37.7)
Chronic dialysis	8 (6.6)
Congestive heart failure	32 (26.2)
Chronic obstructive lung disease	8 (6.6)
Peripheral vascular disease	10 (8.2)
Hyperlipidemia	91 (74.6)
Current Smoker	12 (10.3)
	(*n* = 116)
Medications	
Aspirin	98 (80.3)
Plavix	39 (32.0)
Statins	87 (71.3)
ACE/ARB	65 (53.3)
β-blocker	86 (70.5)
Angiographic Findings	
Number of diseased vessels, median [IQR]	1 [0.0, 2.0]
Multi-vessel coronary disease	34 (27.9)
Percent area stenosis (%), median [IQR]	4.96 [3.78, 6.37]
Gensini Score	26.0 [19.5, 39.0]
Labs, mean ± SD	
Ejection Fraction (%), median [IQR]	57.0 [45.0, 65.0]
	(*n* = 81)
Hemoglobin, (g/dL)	13.17 ± 1.85
	(*n* = 119)
White blood count (1000/mm^3^), median [IQR]	7.83 [6.22, 9.04]
	(*n* = 120)
LDL cholesterol (mg/dL)	86.76 ± 36.23
	(*n* = 98)
Serum creatinine (mg/dL), [median IQR]	1.05 [0.84, 1.30]
Estimated glomerular filtration rate, (mL/min)	68.33 ± 30.41

Baseline characteristics of the study population

*SD* standard deviation, *IQR* inter-quartile range, *Hg* mercury, *min* minute. *ACE*/*ARB* angiotensin converting enzyme inhibitor/angiotensin receptor blocker

### Associations with atherosclerotic burden

Atherosclerotic plaque burden varied widely with a median atherosclerotic burden occupying 5.0 % (inter-quartile range (IQR) 3.8, 6.4) of the total area of the coronary circulation. When analyzed according to quartiles of plaque burden (Table 2), prior MI (*P* = 0.03) and diabetes were more prevalent in those with a higher burden of atherosclerosis (*P* = 0.01) while hemoglobin was lower (*P* = 0.02). Other characteristics aside from alternative measures of plaque burden (e.g., number of diseased vessels, Gensini score) were not significantly different in quartiles of plaque burden. Although there were numerical differences in the level of END, ADMA, TSP, ENG, and ANG across quartiles of plaque burden (Table 3), differences were marginal and did not achieve significance in unadjusted analyses (*P* ≥ 0.18 for all comparisons). Results were similar in analyses using the Gensini score in place of the percent area stenosis (data not shown). There was no evidence of a significant association between factor level and atherosclerotic burden, in analyses adjusted for renal function, acute coronary syndrome on presentation, age, sex, race, diabetes, hyperlipidemia, smoking, hypertension, history of heart failure, and peripheral vascular disease (Table 4).

**Table 2:** Baseline characteristics according to plaque burden measured as total percent area stenosis

Variable (*N* (%))	Q1	Q2	Q3	Q4	*P* value
	2.16–3.78 %	3.86–4.95 %	4.96–6.37 %	6.39–13.2 %	
	(*n* = 31)	(*n* = 30)	(*n* = 31)	(*n* = 30)	
Demographics					
Age (years), mean ± SD	57.03 ± 14.01	62.47 ± 12.47	62.06 ± 9.71	63.97 ± 9.42	0.11
Male	17 (54.8 %)	20 (66.7 %)	23 (74.2 %)	22 (73.3 %)	0.34
Black	3 (9.7 %)	3 (10.0 %)	4 (12.9 %)	1 (3.3 %)	0.61
Blood pressure (mmHg), mean ± SD					
Systolic	123.63 ± 23.04	123.96 ± 25.00	136.52 ± 23.97	127.17 ± 18.05	0.10
Diastolic	72.03 ± 13.63	68.11 ± 12.87	71.71 ± 13.87	68.69 ± 12.93	0.57
Medical history					
Acute coronary syndrome on presentation	8 (25.8 %)	10 (33.3 %)	15 (48.4 %)	10 (33.3 %)	0.30
Prior myocardial infarction	4 (12.9 %)	10 (33.3 %)	8 (25.8 %)	14 (46.7 %)	0.03
Hypertension	20 (64.5 %)	27 (90.0 %)	25 (80.6 %)	23 (76.7 %)	0.11
Diabetes	6 (19.4 %)	8 (26.7 %)	16 (51.6 %)	16 (53.3 %)	0.01
Chronic dialysis	2 (6.5 %)	2 (6.7 %)	3 (9.7 %)	1 (3.3 %)	0.80
Congestive heart failure	7 (22.6 %)	11 (36.7 %)	8 (25.8 %)	6 (20.0 %)	0.47
Chronic obstructive lung disease	2 (6.5 %)	3 (10.0 %)	2 (6.5 %)	1 (3.3 %)	0.78
Peripheral vascular disease	0 (0.0 %)	2 (6.7 %)	4 (12.9 %)	4 (13.3 %)	0.19
Hyperlipidemia	18 (58.1 %)	24 (80.0 %)	26 (83.9 %)	23 (76.7 %)	0.09
Angiographic findings					
Number of diseased vessels, median [QR]	0.0 [0.0, 0.0]	0.0 [0.0, 1.0]	1.0 [1.0, 2.0]	2.0 [2.0, 3.0]	<0.001
Multi-vessel coronary disease	0 (0.0 %)	1 (3.3 %)	9 (29.0 %)	24 (80.0 %)	<0.001
Gensini score, median [IQR]	19.5 [18.5, 21.0]	21.8 [18.5, 27.0]	29.0 [25.5, 44.5]	45.8 [32.5, 65.0]	<0.001
Labs, mean ± SD					
Ejection fraction (%), median [IQR]	60.00 [43.75, 63.63]	60.00 [45.00, 65.00]	53.00 [35.00, 65.00]	55.00 [47.00, 60.00]	0.46
Hemoglobin, (g/dL)	13.55 ± 1.57	13.30 ± 1.23	13.54 ± 1.66	12.28 ± 2.48	0.02
White blood count (1000/mm^3^), median [IQR]	7.72 [6.00, 8.55]	7.22 [6.40, 8.60]	7.95 [5.60, 9.34]	8.26 [6.60, 9.14]	0.14
LDL cholesterol (mg/dL)	101.55 ± 31.19	76.48 ± 30.45	90.04 ± 43.00	81.46 ± 34.67	0.11
Serum creatinine (mg/dL), median [IQR]	1.01 [0.81, 1.30]	1.02 [0.90, 1.22]	1.13 [0.90, 1.41]	1.08 [0.85, 1.24]	0.55
Estimated glomerular filtration rate, (mL/min)	69.01 ± 28.21	70.29 ± 30.70	64.18 ± 33.68	69.96 ± 29.88	0.85

Baseline characteristics of the study population according to percent area stenosis

*SD* standard deviation, *IQR* inter-quartile range, *Hg* mercury, *min* minute

**Table 3:** Association of angiogenesis inhibitor concentrations with plaque burden measured as total percent area stenosis

Factor (median [IQR])	Q1	Q2	Q3	Q4	*P* value
	2.16–3.78 %	3.86–4.95 %	4.96–6.37 %	6.39–13.2 %	
	*n* = 31	*n* = 30	*n* = 31	*n* = 30	
Endostatin (ng/mL)	136.75 [83.51, 198.57]	134.87 [103.91, 191.11]	109.08 [83.17, 184.55]	118.38 [92.03, 150.14]	0.50
ADMA (μmol/L)	0.47 [0.40, 0.54]	0.43 [0.40, 0.51]	0.47 [0.41, 0.50]	0.48 [0.44, 0.56]	0.57
Thrombospondin-2 (pg/mL)	20,900.0 [14,800.0, 26,500.0]	21,000.0 [15,400.0, 33,000.0]	19,100.0 [15,200.0, 26,300.0]	26,000.0 [16,450.0, 33,900.0]	0.45
Endoglin (ng/mL), mean ± SD	4.43 ± 1.49	4.50 ± 1.08	3.91 ± 0.84	4.47 ± 1.23	0.18
Angiopoietin-2 (ng/mL)	2.08 [1.50, 3.11]	2.75 [1.51, 4.48]	2.45 [1.47, 4.86]	2.23 [1.71, 4.44]	0.79

Quartiles of plaque burden and distribution of mean or median values for endostatin (*n* = 115), thrombospondin-2 (*n* = 109), endoglin (*n* = 116), angiopoietin-2 (*n* = 108), and ADMA (asymmetric dimethyl arginine, *n* = 110) concentrations

**Table 4:** Crude and adjusted associations of plaque burden and angiogenesis inhibitor concentration

Factor (median [IQR])	Unadjusted β (95 % CI)	*P* value	Adjusted β (95 % CI)	*P* value
Endostatin (per 100 ng/mL)	−0.16 (−0.04, 0.13)	0.28	0.00 (−0.03, 0.036)	0.97
ADMA (per 0.1 μmol/L)	0.02 (−0.03, 0.08)	0.39	0.03 (−0.02, 0.09)	0.25
Thrombospondin-2 (per 100 pg/mL)	0.02 (0.00, 0.01)	0.37	0.00 (0.00, 0.01)	0.62
Endoglin (per 1 ng/mL)	−0.01 (−0.07, 0.05)	0.81	0.01 (−0.05, 0.07)	0.81
Angiopoietin-2 (per 1 ng/mL)	0.00 (−0.03, 0.02)	0.69	0.01 (–0.02, 0.04)	0.44

Association of inhibitor level with log transformed plaque burden

### Collateral formation

**Fig. 1:**
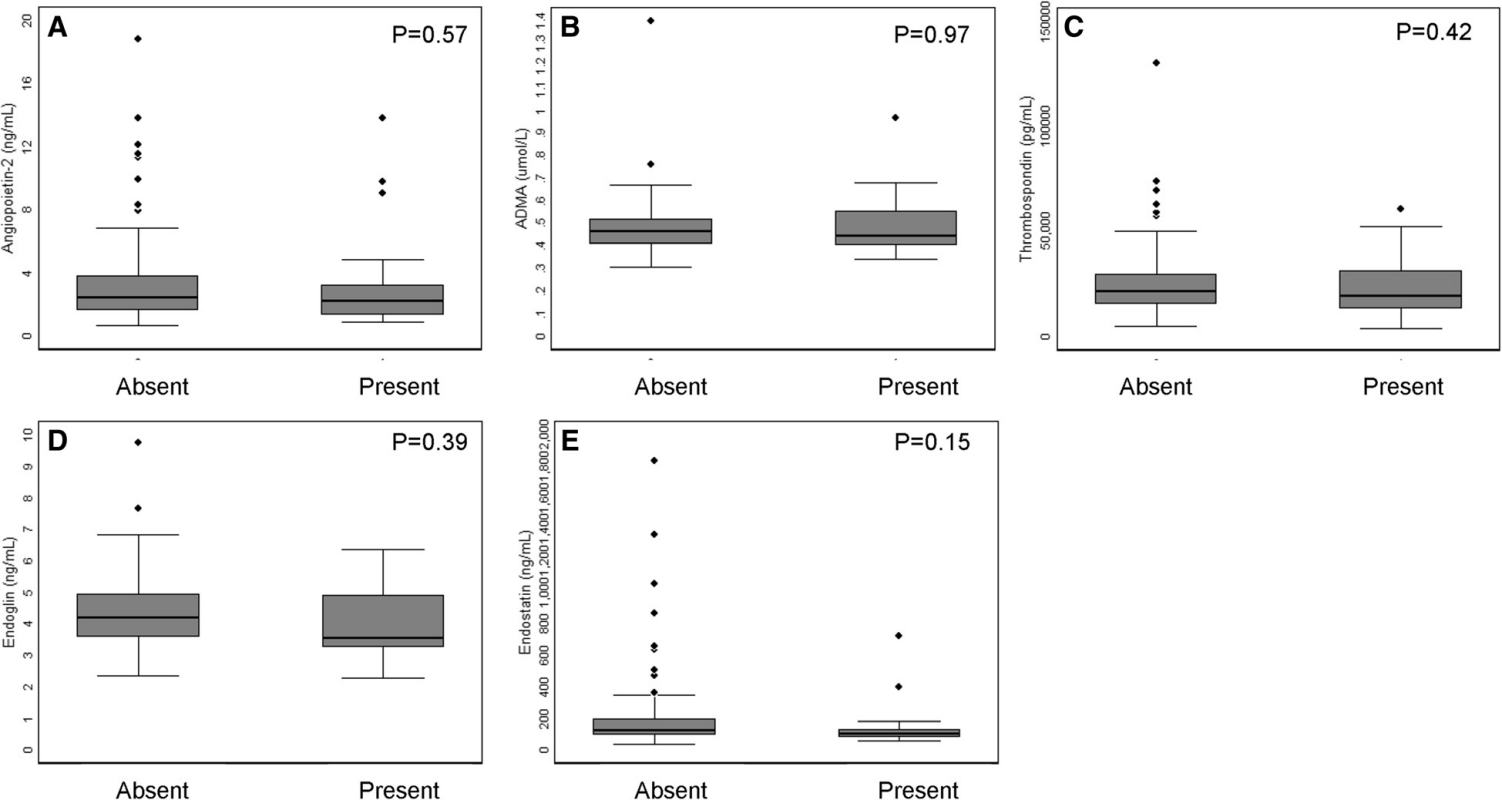
*Box* and *whisker plots* showing factor concentration according to the presence or absence of collaterals. **a** Angiopoietin-2. **b** ADMA. **c** Thrombospondin-2. **d** Endoglin. **e** Endostatin

Collateral vessels were infrequent and were found in only 20 (16.4 %) subjects. A history of prior MI (*P* = 0.03) and peripheral vascular disease (*P* = 0.04) were more frequent among those with compared to those without collaterals (Table 5). The number of diseased vessels was higher, multi-vessel disease was more common, and indices of plaque burden were significantly greater in individuals with compared to those without collaterals. END (126.7 ng/mL: IQR [95.0, 198.6] vs 105.7 ng/mL: IQR [80.4, 131.1]), ENG (4.2 ng/mL: IQR [3.6, 5.0] vs. 3.5 ng/mL: IQR [3.2, 4.9]). ANG (2.2 ng/mL: IQR [1.6, 3.8] vs. 2.2 ng/mL: IQR [1.3, 3.2]), ADMA (0.47 μmol/L: IQR [0.41, 0.52] vs. 0.45 μmol/L: IQR [0.41, 0.56]), and TSP (21650.0 pg/mL: IQR [15400.0, 29850.0] vs. 19,100 pg/mL: IQR [13400, 31350]) concentrations were not significantly different in individuals without compared to individuals with visible collateral formation (Fig. 1). Inhibitor concentration was not associated with collateral formation in either crude or adjusted analyses (Table 6). The results were similar in sensitivity analyses in which individual vessels rather than individual patients severed as the unit of analysis (data not shown).

**Table 5:** Baseline characteristics and collateral formation

Variable (*N* (%))	Collaterals absent	Collaterals present	*P* value
	(*n* = 102)	(*n* = 20)	
Demographics			
Age (years), mean ± SD	61.38 ± 11.86	61.20 ± 11.28	0.95
Male	67 (65.7 %)	15 (75.0 %)	0.42
Black	11 (10.8 %)	0 (0.0 %)	0.12
Blood pressure (mmHg), mean ± SD			
Systolic	129.77 ± 23.44	119.15 ± 18.88	0.06
Diastolic	70.40 ± 13.88	69.20 ± 10.13	0.72
Medical History			
Acute coronary syndrome on presentation	34 (33.3 %)	9 (45.0 %)	0.32
Prior myocardial infarction	26 (25.5 %)	10 (50.0 %)	0.03
Hypertension	78 (76.5 %)	17 (85.0 %)	0.41
Diabetes	38 (37.3 %)	8 (40.0 %)	0.82
Chronic dialysis	8 (7.8 %)	0 (0.0 %)	0.20
Congestive heart failure	26 (25.5 %)	6 (30.0 %)	0.68
Chronic obstructive lung disease	8 (7.8 %)	0 (0.0 %)	0.20
Peripheral vascular disease	6 (5.9 %)	4 (20.0 %)	0.04
Hyperlipidemia	75 (73.5 %)	16 (80.0 %)	0.54
Angiographic Findings			
Number of diseased vessels, median [IQR]	1.0 [0.0, 1.0]	2.0 [1.0, 2.5]	<0.001
Multi-vessel coronary disease	20 (19.6 %)	14 (70.0 %)	<0.001
Gensini score, median [IQR]	22.25 [19.50, 30.50]	66.50 [56.25, 92.00]	<0.001
Percent area stenosis (%), median [IQR]	4.7 [3.6, 6.1]	0.7 [5.0,9.0]	<0.001
Labs, mean ± SD			
Ejection fraction (%), median [IQR]	57.5 [45.0, 65.0]	55.0 [38.0, 64.0]	0.53
Hemoglobin, (g/dL)	13.25 ± 1.82	12.76 ± 2.01	0.28
White blood count (1000/mm^3^), median [IQR]	7.66 [6.13, 8.93]	8.17 [6.96, 9.40]	0.22
LDL cholesterol (mg/dL)	88.55 ± 33.58	79.75 ± 45.45	0.34
Serum creatinine (mg/dL), median [IQR]	1.05 [0.83, 1.30]	1.05 [0.89, 1.41]	0.61
Estimated glomerular filtration rate, (mL/min)	68.15 ± 30.43	69.28 ± 31.03	0.88

**Table 6:** Crude and adjusted associations of angiogenesis inhibitor concentration with collateral formation

Factor (median [IQR])	Crude odds ratio (95 % CI)	*P* value	Adjusted odds ratio (95 % CI)	*P* value
Endostatin (per 100 ng/mL)	0.88 (0.64, 1.21)	0.44	0.91 (0.65, 1.28)	0.61
ADMA (per 0.1 μmol/L)	1.10 (0.79, 1.54)	0.56	1.04 (0.73, 1.50)	0.81
Thrombospondin-2 (per 1000 pg/mL)	0.99 (0.96, 1.02)	0.59	0.97 (0.93, 1.02)	0.21
Endoglin (per 1 ng/mL)	0.82 (0.52, 1.29)	0.39	0.76 (0.46, 1.26)	0.28
Angiopoietin-2 (per 1 ng/mL)	1.01 (0.87, 1.18)	0.88	1.05 (0.88, 1.25)	0.60

Adjusted for acute coronary syndrome and percent area stenosis on angiography

## Discussion

We analyzed atherosclerotic plaque burden and coronary collateral formation using quantitative coronary angiography and assessed the associations of angiogenesis and NO inhibitors with coronary artery disease in individuals undergoing non-emergent angiography. Although there were small, numerical differences in the concentrations of several factors, the systemic concentrations of ADMA, ENG, END, TSP, or ANG were not significantly associated with total plaque burden or presence of collaterals in either crude or adjusted analyses.

Endothelial dysfunction and plaque angiogenesis have been implicated in the pathogenesis of coronary artery disease suggesting a key role for NO homeostasis and angiogenesis inhibitors in the development of human atherosclerosis [3–6]. In addition to this direct role, NO bioavailability and inhibitors of angiogenesis may modify risks associated with the presence of coronary disease by inhibiting the development of collateral circulations that reduce myocardial damage and diminish morbidity and mortality from myocardial ischemia [15–19]. As a result, there has been a great interest in determining whether NO or elated inhibitors of angiogenesis are associated with coronary atherosclerosis.

Although direct measurement of NO in humans is technically challenging, ADMA, a competitive inhibitor of NO synthase [14], has been widely studied. ADMA has been implicated as an independent risk factor for both all-cause mortality and cardiovascular events [20, 21], and it has been associated with the presence of atherosclerosis in some but not all studies [22, 23].

Relatively few studies have examined associations with coronary atherosclerosis. However, in a recent study of 152 non-diabetic men with obstructive coronary disease (≥1 vessel with 70 % atherosclerosis), ADMA was correlated with the proportion of the length of the coronary tree occupied by luminal irregularities, but not with number of diseased vessels or the Gensini score [24]. Another study of 997 participants found that ADMA concentration was higher in individuals with ≥50 % coronary stenosis of a coronary artery compared to those without significant atherosclerosis. However, only unadjusted analyses were performed and adjustment for potential confounders was not performed [25]. Conversely, another study including 1364 patients with stable angina reported that ADMA levels were actually significantly lower in subjects with compared to those without obstructive coronary disease, although there were no differences in the prevalence of three-vessel disease [26]. The results were similar in a second, large study including more than 3000 German subjects [27].

The association of collateral formation and ADMA has been less well studied. However, in a small study of 61 patients, ADMA concentration was not associated with collateral formation [28]. In contrast, Kocaman studied 74 individuals and found that individuals with collateral development had lower plasma ADMA (0.41+/−0.25 μmol/L) compared to those without collateral formation (0.70+/−0.23 μmol/L, *P* = 0.001) [29].

Our analysis is consistent with the largest prior studies which similarly failed to demonstrate an association between ADMA level and coronary atherosclerosis or collateral formation [27, 26]. Our study extends these observations by analyzing both aspects of coronary disease within a single cohort, by adjusting for relevant risk factors in multi-variable analyses, and by the use of digital angiography and a fully quantitative method for assessing atherosclerotic burden thereby permitting more accurate measurement. Although, as noted above, some prior studies have identified associations of ADMA with atherosclerosis [22, 24, 25], the failure to confirm an association of ADMA with a quantitative measure of plaque burden or with collateral formation suggests that the associations of ADMA with the risk of all-cause and cardiovascular mortality [20, 21] are not mediated primarily through the development or extension of atherosclerotic plaques. Whether ADMA impacts plaque vulnerability is unknown but further study appears warranted in light of our data.

We also studied additional angiogenesis inhibitors—specifically END, TSP, ANG, and ENG, each of which has been shown to be induced by or to modulate the response to changes in NO bioavailability [7–13, 30]—with plaque burden and collateral formation. Relatively few studies have investigated associations of END with coronary atherosclerosis, although myocardial RNA expression of END was negatively correlated with the Rentrop score in one study [31]. Similarly, the difference between coronary sinus and left ventricular END concentration was 1.9-fold higher in individuals without compared to those with collaterals in a study of 72 patients [32]. Finally, in a study of 39 patients, endostatin levels in pericardial fluid were inversely correlated with collateral formation [33]. However, circulating END was not correlated with collateral formation in an angiographic study of 101 patients [34] or in a second study of 218 patients [35]. To our knowledge, the association of circulating END with plaque burden has not been directly reported, although coronary sinus END concentration was 1.6-fold higher in individuals with coronary artery disease compared to those with atypical chest pain [32].

Elevated levels of TSP [36], ANG [37], and ENG [38] are associated with increased risks of cardiovascular mortality. An autopsy study of nine patients demonstrated an increased frequency of ENG expression in coronary lesions with intra-plaque hemorrhage—a marker of plaque vulnerability [39]—but to our knowledge, associations of ENG with plaque burden or collateral formation have not been previously published. Similarly, TSP polymorphisms have been linked to the risk of MI [40] and the presence of coronary atherosclerosis [41], but to our knowledge, the association of circulating concentrations with plaque burden or collateralization has not been previously reported. Lastly, despite experimental models convincingly linking ANG with myocardial vascular supply [42], we are aware of only a single, small study investigating the association between ANG and human coronary artery disease in which ANG concentration was not associated with atherosclerosis (as assessed by maximal percent stenosis) or with collateral supply [43].

Our study extends prior reports by assessing associations of END, ENG, TSP, and ANG with both plaque burden and collateral supply in a single cohort. As noted above, the use of QCA techniques and a fully quantitative measurement of plaque burden represent other unique features of our study. The larger sample size of our study compared to most prior analyses of these factors allowed us to correct for potential confounders, but we did not identify any significant associations in either crude or adjusted models. Our findings suggest that associations of circulating END, ENG, TSP, and ANG with cardiovascular mortality are unlikely to be mediated via direct effects on coronary plaque formation or by inhibition of collateral formation. Whether local concentrations, myocardial tissue expression, or intra-plaque expression plays a more important role or whether these factors could impact plaque vulnerability is unknown. Additional studies in larger cohorts with the ability to analyze tissue and pericardial expression are clearly warranted.

Our findings should be interpreted within the context of our study design. Power to rule out small differences in concentration between groups was limited. Our findings did not particularly suggest trends across groups in measured concentration although there were non-significant differences across groups in END concentration. Larger studies are needed to definitively rule out differences. The sample sizes used allowed us to correct for the most important factors in multi-variable models but did not permit simultaneous adjustment for all potential confounders—particularly in the analysis of collaterals, where the overall number of collaterals was low. Lastly, our subjects came from a single center, and our results may not be fully generalizable to populations with different genetic backgrounds or indications for angiography. Lastly, we were unable to measure tissue concentrations or localized blood concentrations for the measured factors. Thus, larger, multi-center studies with the ability to measure tissue or local factor concentrations should be considered.

## Conclusions

In conclusion, we used QCA to analyze associations of a broad panel of related NO and angiogenesis inhibitors with plaque burden and collateral formation using quantitative coronary angiography. We did not find any significant associations between the severity of atherosclerosis or collateral formation and the circulating concentrations of ADMA, END, ENG, ANG, or TSP. Our findings suggest that the circulating concentration of these factors may not be important determinants underlying the development of atherosclerosis or the formation of coronary collaterals. Further studies to confirm our findings and to investigate the impact of tissue or local concentrations of angiogenesis and NO inhibitors on coronary atherosclerosis and plaque vulnerability should be considered.

## Methods

### Ethical approvals

This study was approved by the Partners Human Research Committee and institutional review board of the Beth Israel Deaconess Medical Center (both in Boston, Massachusetts). All research was in compliance with relevant regulation and the Helsinki declaration. Written informed consent for participation, phlebotomy, and analysis of biologic samples and data was obtained from all participating subjects.

### Study population

Individuals between 18–80 years of age were recruited from the cardiac catheterization suite at a single center. Subjects with a history of coronary bypass surgery or undergoing emergency angioplasty in the setting of acute myocardial infarction (MI) were excluded as were individuals with acute kidney injury, those receiving anti-angiogenic or immunosuppressive medications, and individuals with a history of thoracic radiation or cancer. Subjects underwent a brief interview and physical exam, and additional clinical data were extracted by chart review. Serum and plasma were collected before angiography, centrifuged within 20 min at 1000×*g*, and stored at −80 °C. Serum creatinine and blood counts were measured on the day of the procedure in the clinical laboratory, and cholesterol measurements within 6 months prior to enrollment were recorded. Glomerular filtration rate (eGFR) was calculated using the pre-procedure creatinine and the modified MDRD equation [44].

### Measurement of circulating factors

Endoglin (ENG), serum END, and ANG were measured using Quantikine® ELISA (R&D Systems) with intra- and inter-assay coefficient of variations (CVs) <7.0 and <13.2 %, respectively. A Luminex assay (R&D Systems) was used to measure TSP with CVs <8.7 and 16.4 %, respectively. Plasma ADMA was measured by HPLC according to the method of Teerlink [45] (intra and inter-assay CV <3.5 %).

### Angiographic analysis

All coronary angiograms were analyzed by a single reader using standard QCA software (Cardiology Medis System, version 5.1, Nuenen, The Netherlands). Foreshortening and measurement variability were minimized by utilizing a standard sequence of angiographic views [46] and by calibrating sizes against catheter dimensions [47]. Percent stenosis was recorded only when ≥20 %—the lower limit of reliable discrimination. Arteries with diameters <1.75 mm were not assessed because these segments were considered too small for accurate QCA.

**Fig. 2:**
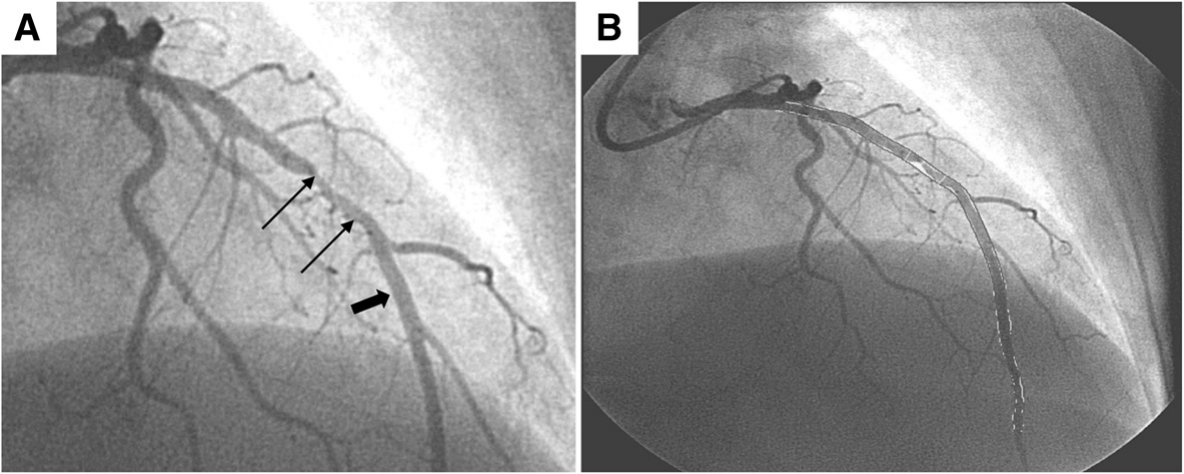
Images from QCA of coronary angiogram before (**a**) and after (**b**) digital processing. **a** *Thick arrow* shows typical area without significant luminal irregularities. *Thin arrows* point to luminal narrowing and irregularity. **b** Post-processing images demonstrating the vessel wall outlined in *black* and areas with luminal irregularity traced in *white*

Two-dimensional plaque area was quantitatively measured within each coronary artery segment (Fig. 2). The differences in the total length and evaluable portion of individual coronary arteries were standardized by using the percent area stenosis—defined as the two-dimensional area occupied by luminal plaque divided by the total luminal area of all evaluated coronary segments—as the primary outcome. The Gensini score [48] was assessed in a sensitivity analysis.

Coronary collateral formation was assessed using the Rentrop classification [49]—a four-point, semi-quantitative system in which scores are scaled from 0 to 3: 0 (no visible collaterals), 1 (side branch filling via collateral channels), 2 (partial filling of the epicardial arterial segment via collaterals), and 3 (complete filling through dilated collaterals).

### Statistical analysis

Data are presented according to their distribution as mean ± standard deviation (SD), *n* (%), or median (IQR). In unadjusted analyses, differences were compared using ANOVA or Kruskal-Wallis tests for continuous data and Chi-square tests for count data. Adjusted outcomes were assessed using multi-variable linear regression (percent area stenosis, Gensini score) or logistic regression (collateral formation) with logarithmic transformation of outcome variables as needed to preserve normality. In a secondary analysis using collateral supply to each individual coronary artery as the unit of analysis, we used robust standard errors and clustering by individual to account for the correlation in the propensity to form collaterals between multiple arteries (left, right, and circumflex coronary arteries) within a given individual. Model fit was tested graphically, by inspecting residuals and by testing model specification. The analyses were performed in STATA 13.0 (STATA Corp.) with *P* < 0.05 considered significant.

## Abbreviations

ACE/ARB: angiotensin converting enzyme inhibitor or angiotensin receptor blocker
ADMA: asymmetric dimethyl arginine
ANG: angiopoietin-2
CKD: chronic kidney disease
END: endostatin
ENG: endoglin
IQR: inter-quartile range
MI: myocardial infarction
NO: nitric oxide
OR: odds ratio
QCA: quantitative coronary angiography
TSP: thrombospondin
